# Factors associated with antiretroviral treatment failure among people living with HIV on antiretroviral therapy in resource-poor settings: a systematic review and metaanalysis

**DOI:** 10.1186/s13643-020-01524-1

**Published:** 2020-12-12

**Authors:** Yishak Lailulo, Marcel Kitenge, Shahista Jaffer, Omololu Aluko, Peter Suwirakwenda Nyasulu

**Affiliations:** 1 Division of Epidemiology and Biostatistics, Department of Global Health, Faculty of Medicine and Health Sciences, Stellenbosch University, Cape Town, South Africa; 2 Médecins Sans Frontières (MSF), Eshowe, KwaZulu Natal South Africa; 3 Division of Epidemiology & Biostatistics, School of Public Health, Faculty of Health Sciences, University of the Witwatersrand, Johannesburg, South Africa

**Keywords:** HIV, ART, Immunological failure, Virological failure, Clinical failure, Poor outcome

## Abstract

**Background:**

Despite the increase in the number of people accessing antiretroviral therapy (ART), there is limited data regarding treatment failure and its related factors among HIV-positive individuals enrolled in HIV care in resource-poor settings. This review aimed to identify factors associated with antiretroviral treatment failure among individuals living with HIV on ART in resource-poor settings.

**Methods:**

We conducted a comprehensive search on MEDLINE (PubMed), Excerpta Medica Database (EMBASE), Cochrane Central Register of Controlled Trials (CENTRAL), World Health Organization’s (WHO’s) library database, and Latin American and Caribbean Health Sciences Literature (LILACS). We included observational studies (cohort, case-control, and cross-sectional studies) where adolescents and adults living with HIV were on antiretroviral treatment regardless of the ART regimen. The primary outcomes of interest were immunological, virological, and clinical failure. Some of the secondary outcomes were mm^3^ opportunistic infections, WHO clinical stage, and socio-demographic factors. We screened titles, abstracts, and the full texts of relevant articles in duplicate. Disagreements were resolved by consensus. We analyzed the data by doing a meta-analysis to pool the results for each outcome of interest.

**Results:**

Antiretroviral failure was nearly 6 times higher among patients who had poor adherence to treatment as compared to patients with a good treatment adherence (OR = 5.90, 95% CI 3.50, 9.94, moderate strength of evidence). The likelihood of the treatment failure was almost 5 times higher among patients with CD4 < 200 cells/mm^3^ compared to those with CD4 ≥ 200 CD4 cells/mm^3^ (OR = 4.82, 95% CI 2.44, 9.52, low strength of evidence). This result shows that poor adherence and CD4 count below < 200 cells/mm^3^ are significantly associated with treatment failure among HIV-positive patients on ART in a resource-limited setting.

**Conclusion:**

This review highlights that low CD4 counts and poor adherence to ART were associated to ART treatment failure. There is a need for healthcare workers and HIV program implementers to focus on patients who have these characteristics in order to prevent ART treatment failure.

**Systematic review registration:**

The systematic review protocol was registered with the International Prospective Register of Systematic Reviews (PROSPERO), registration number: 2019 CRD42019136538.

## Background

Human immunodeficiency virus (HIV) infections are a major global public health concern. In 2019, an estimated 38 million people were living with HIV infection (PLWH) [1]. With new infections, an estimated 1.7 million people became newly infected with HIV in 2019. Sub-Saharan Africa (SSA) remains the most affected region in the world, with about 20.7 million prevalent cases and 730,000 new infections were recorded in 2019, seconded by Asia and the Pacific region with 5.8 million prevalent cases [1]. Although Southern Africa is home to less than 1% of the global population, the region has more than a fourth of all HIV infection in the world, with 300,000 acquired immune deficiency syndrome (AIDS)-related deaths registered in the same year in SSA [1].

Although anti-retroviral therapy (ART) coverage in this region has rapidly increased over the past decade [2]. The greatest gains in access to ART occurred in SSA [3]. In 2019, only 15 million (73%) PLHIV in the region were accessing ART, while 3.5 million (60%) in Asia and the Pacific region [1]. Increasing the use of ART has contributed to a prominent decline in HIV-associated morbidity and death/mortality in SSA [2]. United Nations program on HIV/AIDS (UNAIDS) has suggested universal targets for the year 2020 (90-90-90), which means diagnosing 90% of all PLHIV who should know their status (PLHIV), initiating antiretroviral treatment (ART) for 90% of those diagnosed with HIV infection, and attaining an undetectable viral load in 90% of those on ART [4]. Significant progress has been made in achieving that goal. Globally, PLWH accessing ART has increased from 21.7 million in 2015 to 25.4 million in 2019, an increase from 45 to 67% of all PLHIV [3, 5].

### Antiretroviral treatment failure

Patients with ART failure are increasingly encountered in resource-limited settings, while recent estimates suggest only 2% of those currently on ART are on second-line [6], a far greater number is likely to be failing virologically but have not switched to an alternative regimen. Furthermore, an increase in the coverage of ART use among PLHIV, which has resulted in an increase in the number of individuals failing first-line ART, and therefore, the magnitude increases with prolonged use of ART. The WHO predicted earlier on that 500,000 and 800,000 PLWH on the first-line combination of ART will require a switch to the second-line therapy by 2010 [2]. However, the burden of treatment failure is not well-documented, while there is a large scale of ARV in resource-limited countries. Meta-analysis data showed that the rate of the treatment failure for the first-line was 6.08% globally; however, the study noted a substantial heterogeneity across regions with 7.10% in Africa and 2.55% in Asia [3].

A retrospective cohort study done in South Africa found that among patients on non-nucleotide reverse transcriptase inhibitor (NNRTI)-based ART, after a median of 15 months on ART treatment, 19% had failed virologically and immunologically [6]. Studies in East Africa have shown a high prevalence of immunologic failure ranging from 8 to 57% among clients on the first-line ART [7–9].

Treatment failure is typically measured in three ways in poor-resource settings: (i) clinically, as evidenced by disease progression; (ii) immunologically, as evidenced by trends in CD4 counts over time; and (iii) virologically, as evidenced by measurement of HIV RNA levels. In 2013, WHO recommended viral load testing as the preferred monitoring approach to diagnose and to confirm ARV treatment failure [10].

### Factors associated with treatment failure

Earlier studies have emphasized a number of factors that may be associated with virological suppression in ART; these are reasons for testing: routine testing, suspected treatment failure, and repeat testers after suspected failure [9–11]. While a significant number of studies have found that treatment failure is significantly associated with young age, unsatisfactory adherence, low hemoglobin, history of lost to follow-up, being male and educational status, and treatment regimen [12–14], some studies have recognized low baseline CD4 cell count, rate of CD4 decline, prior exposure to ART and treatment interruptions, and non-adherence as determinants of treatment failure [15, 16].

In 2016, WHO most recent guideline defined a clinical failure as a new or recurrent clinical event indicating severe immunodeficiency (WHO clinical stage 4 condition) after 6 months of effective treatment. Immunological failure is defined as CD4 count at or below 250 cells/mm^3^ following a clinical failure or persistent CD4 levels below 100 cells/mm^3^, and virological failure is defined as viral load above 1000 copies/mL based on two consecutive viral load measurements in 3 months, with adherence support following the first viral load test [17]. The results from a previous study have confirmed that low baseline CD4 cell count, particularly < 100 cells/mm^3^, and history of loss to follow-up are risk factors for immunological discordance [18]. Independent risk factors associated with virological failure were being followed-up at the semirural center, having experienced unstructured treatment interruptions, and having low CD4 counts at enrolment [19].

Gender, time on ART, baseline CD4 T cell count, WHO stage, ART regimen, adherence, and TB co-infection were associated with viral suppression [20]. The history of the antiretroviral use before starting ART, change of antiretroviral therapy due to toxicity, opportunistic infections while on ART treatment, level of CD4 + lymphocytes below 100 cells/ml at start of ART, adherence, and clinical stage were independently associated with virological failure [21]. Age younger than 40 years was also associated with virologic failure [22]. The relative contribution of the main predictors to virological failure may differ across settings and population groups and context. Thus, specific data are critical to the carrying out of corrective measures.

### Importance of the review

Viral load testing provides early and accurate indications of the treatment failure and the need to switch from the first-line to second-line drugs, thereby reducing the accumulation of the drug-resistant mutations and improving clinical outcomes [23].

However, regular access to routine viral load testing remains a challenge due to the high cost. In such a situation, clinical and immunological monitoring is used for detecting treatment failure [24–27]. The number of people accessing ART has significantly increased in many poor resource settings [28]. Hence, it is significant to sustain treatment success and limit the development of treatment failure. For the timely detection of treatment failure, WHO reconfirmed the use of viral load testing as the gold standard test to monitor patients’ response to ART [29]. Where the viral load is not routinely available, CD4 count and clinical monitoring should be used to diagnose treatment failure. In spite of a large number of patients receiving ARTs in low- and middle-income countries (LMICs) and poor settings, there are few reports on ART outcomes in these settings. Identifying baseline predictors of the first-line ART outcome among PLWH on ART in LMICs where access to viral load testing is limited is of paramount importance.

The technique and accuracy of identifying treatment failure in poor settings are important but challenging. Delayed detection of ART failure may increase drug toxicity may lead to the increase of drug resistance related with mutations (further controlling treatment choices) and may result in increased morbidity and mortality. Early detection of treatment failure is crucial to ensure the effectiveness of the first-line therapy [6].

The main objective of this review was to identify factors associated with antiretroviral treatment failure among PLWH on ART in resource-poor settings.

## Objective

### Primary objective

The primary objective of the study was to determine the clinical, immunological, and virological factors associated with antiretroviral treatment failure among PLWH in resource-poor settings.

### Secondary objective

The secondary objective of the study is to identify the socio-demographic and economic factors associated with antiretroviral treatment failure among PLWH among PLWH in resource-poor settings.

## Methods

The methods of this systematic review and meta-analysis were reported as per the Preferred Reporting Items for Systematic Review and Meta-Analysis Protocols (PRISMA-P) checklist [30]. We registered the protocol for this systematic review on the International Prospective Register of Systematic Reviews (PROSPERO) with a registration number: CRD42019136538.

### Criteria for considering studies for review

#### Types of studies

We included all types of observational studies including prospective/retrospective or ambi-directional cohort studies, case-control studies, population-based/nested or hospital-based case-control studies, and cross-sectional studies. Interventional studies were excluded from this review.

#### Types of participants

Adolescents and adults living with HIV who were on ART for ≥ 6 months, regardless of the regimen. Only participants with documented baseline CD4 and VL were considered for this systematic review.

### Type of outcome

#### Primary outcome

Treatment failure was defined as follows:

#### Virological failure

Virological failure is defined as a plasma viral load above 1000 copies/ml based on two consecutive viral load measurements after 3 months, with adherence support. A viral load test is a measurement of the amount of HIV in a sample of the blood. This is usually reported as the number of copies per milliliter (copies/mm^3^) [17].

#### Immunological failure

Immunological failure is defined as a fall in CD4 count to the baseline (or below) or persistent CD4 levels below 100 cells/mm^3^. The CD4 lymphocyte count is an excellent indicator of how healthy the immune system is. These are a type of white blood cells, called T cells, which move throughout the human body to find and destroy bacteria, viruses, and other invading germs. The CD4 cell count is indicated in cells per mm^3^, and it is measured by taking a blood sample [17].

#### Clinical failure

Clinical failure is defined as the occurrence of new opportunistic infections (excluding immune reconstitution inflammatory syndrome [IRIS]) and/or other clinical evidence of HIV disease progression during therapy. AIDS-defining illnesses (opportunistic infections) are those which the Centers for Disease Control and Prevention (CDC) have classified as being directly associated with advanced HIV infection. We considered the common diseases, which are pneumonia, TB, lymphoma, and cryptococcosis [17].

#### Secondary outcome

Secondary outcomes for this study are all the predictors’ variables that contribute to treatment failure. The following information was collected if measured at baseline: CD4 cells (cells/mm^3^), viral load (copies/ml), WHO clinical, tuberculosis, opportunistic infection, treatment regimen (NRTI or NNRTI), BMI, weight, study site (rural versus urban), gender, age, educational status, employment status, marital status, and spouse HIV sero-status.

### Inclusion and exclusion criteria

#### Included studies

Participants in the study were (1) those who had been on ART for ≥ 6 months and (2) those who had documented CD4 cell count and viral load measurement at baseline and 6 months.

#### Excluded studies

All studies with participants who had pregnancy history the past 6 months while on treatment and at 6 months’ visit or had missing values of CD4 cell count and viral load at baseline and 6 months’ visit were excluded.

#### Search methods for identification of the studies

We conducted a comprehensive search on 5 databases from December 1, 2000, to November 2019. With assistance from an information specialist, we searched in the following databases: MEDLINE (Pubmed), EMBASE (OVID), LILACS (BIREME), Science Citation Index Expanded (SCI-EXPANDED, Web of Science), Social Sciences citation index (SSCI, Web of Science), Conference Proceedings Citation Index-Social Science & Humanities (CPCI-SSH, Web of Science), and Cinahl (EBSCOHost). A detailed search strategy is provided in Appendix 1. A hand search of citations from selected studies was conducted to identify additional studies missing from the original electronic searches.

#### Screening and assessments of study eligibility

All potential studies were imported into Covidence (Covidence systematic review software, Veritas Health Innovation, Melbourne, Australia), and two review authors (YL and SJ) independently screened the titles and abstracts. Both authors also assessed full-text eligibility.

All published full-text articles, abstracts, and brief reports were included, and provided/available complete data were elicited from them. The disagreements between the two authors who assessed study eligibility were resolved by discussion and consensus.

#### Data extraction, management, and analysis

Data from the full-text articles were extracted by two independent review authors (YL, SJ) using a standardized pre-piloted data extraction form. A third reviewer (MK, PN) checked whether the extracted data were correct. Extracted data were categorized into four main headings: general information, socio-demographic and economic characteristics of participants, and clinical and immunological information of the participant. In case of missing information, we clarified the conducted study or the studies that had relevant data, which were not reported in the published manuscript, and we contacted the authors for additional information.

#### Risk of bias and quality of evidence

Two authors independently assessed the risk of bias in each study by examining the study population, study attrition, prognostic factor measurement, outcomes measurement, study confounding, and statistical reporting (YL and OA). They coded studies as at high, medium, low, or unclear risk of bias for each of these features using the Quality in Prognosis Studies tool (QUIPS tool) [31]. Finally, we assessed the quality of the evidence using the Grading of Recommendations Assessment Development and Evaluations (GRADE) approach using the five criteria of the GRADE system.

#### Statistical analysis

For the studies that were relatively homogeneous in terms of methodology and outcomes, a meta-analysis of the data was performed. Sufficiently, similar data was pooled using the inverse variance approach to accommodate crude and adjusted odds ratios, where possible. Additionally, the meta-analysis was summarized using pooled estimates, the 95% confidence interval, and the between-study variance was estimated using Tau^2^. We extracted all unadjusted and adjusted measures of the association from all included studies and converted effect sizes as necessary to possible selection bias, thus allowing us to use the data from as many studies as possible. We anticipated that results from multivariate analyses would have been reported as odds ratios (ORs), risk ratios (RRs), and hazard ratios (HRs), if so, we would use ORs as the common measure of the association, using RRs and HRs to estimates ORs at a particular time point [32]. Furthermore, measures of effect were analyzed using RevMan statistical software for systematic reviews. Statistical heterogeneity was quantified using the *I*^2^ statistic [33]. If the *I*^2^ statistic is high (75 to 100%—as suggested by Higgins et al.) indicating high heterogeneity [33], a random effect model was used.

## Results

### PRISMA flow chart

**Fig. 1:**
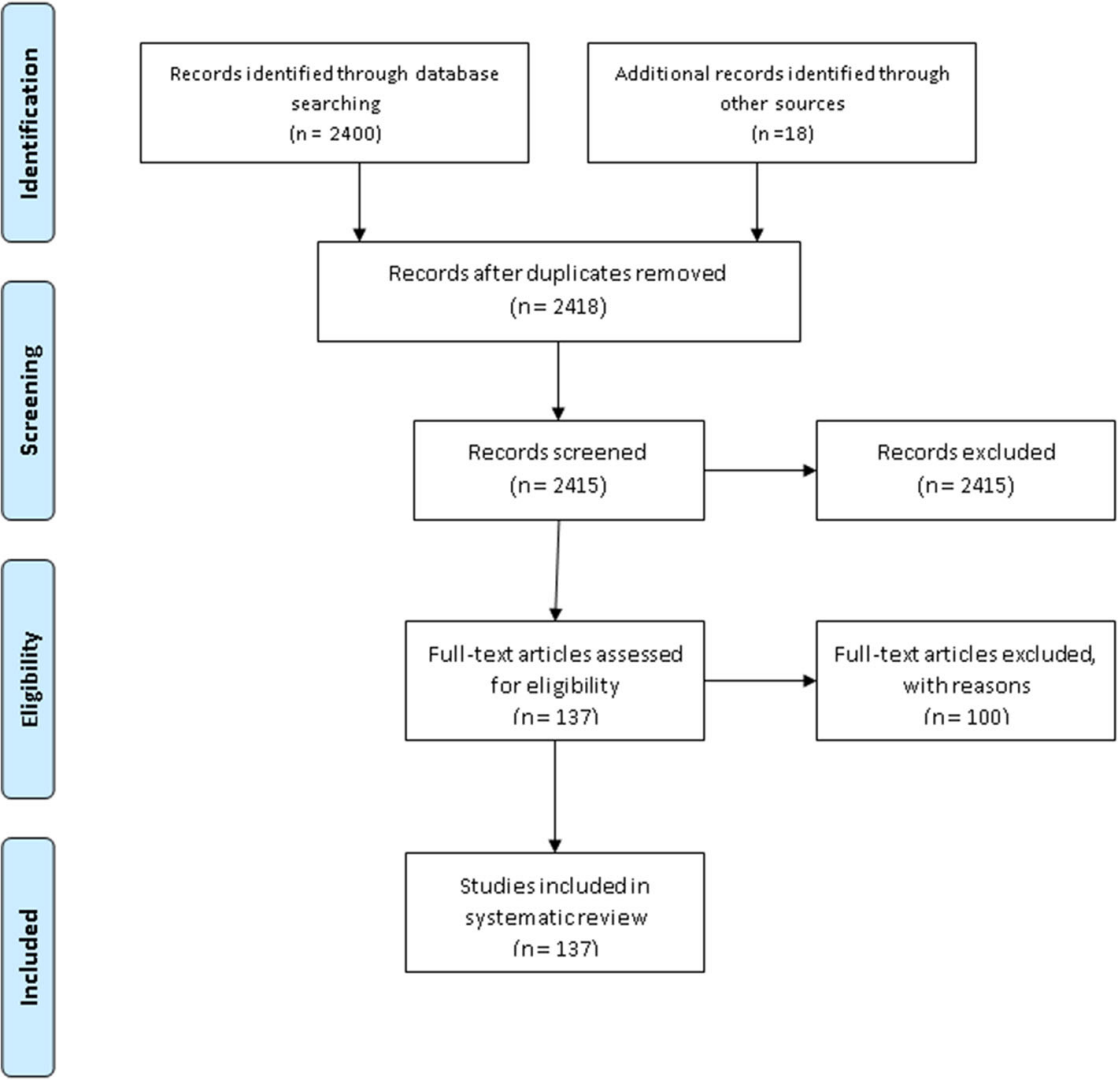
PRISMA flowchart of included studies

We retrieved 2418 articles regarding treatment failure among ART users in poor resource setting as identified in MEDLINE (PubMed); EMBASE (OVID); LILACS (BIREME); Science Citation Index Expanded (SCI-EXPANDED, Web of Science), Social Sciences citation index (SSCI, Web of Science), and Conference Proceedings Citation Index-Social Science & Humanities (CPCI-SSH, Web of Science), and CINAHL (EBSCOHost). These are shown in Fig. 1.

Of these initial articles, 3 articles were duplicates; 2158 articles were excluded after reviewing their titles and abstracts and confirmed irrelevant to this review. Thus, 237 potential full-text articles were assessed for eligibility, which resulted in further exclusion of 100 articles. 57 had wrong outcomes, 19 assessed HIV drug-resistant mutations, 12 had the wrong study design, 7 had a wrong patient population, 2 were not in English 1 and was a duplicate, 1 had a wrong setting, and 1 was pediatric population. Finally, 137 studies met the eligibility criteria. These are shown in Table 1.

**Table 1:** Characteristics of included studies

References	Year of publication	Study design	Country	Patients groups	ART used	Sample size	Number of Treatment failure
Babo et al. [34]	2017	Case-control study	Ethiopia	Adult	Stavudine vs. Zidovudine Nevirapine vs. Efavirenz	307	230
Bayu et al. [35]	2017	Case-control study	Ethiopia	Adults aged ≥ 15 years	D4T-based AZT-based TDF-based	306	160
Bilcha et al. [36]	2019	Retrospective cohort study	Ethiopia	Adult	Nevirapine-based Efavirenz-based	396	47
Bisson et al. [37]	2008	Case-control study	Botswana	Adults older than 18 years	NR	302	247
Fatti et al. [38]	2019	Prospective cohort study	South Africa	Adults aged ≥ 18 years	NRTI and NNRTI	1901	60
Ford et al. [39]	2010	Observational cohort	South Africa	Adult	EFV, NVP, and other	207	32
Gunda et al. [40]	2019	Case-control study	Tanzania	Adult	AZT/3TC/EFV, AZT/3TC/NVP, D4T/3TC/NVP, TDF/3TC/EFV	197	24
Haile et al. [41]	2016	Retrospective cohort study	Ethiopia	Adult (≥ 15 years old)	1a(d4T + 3TC + NVP), 1b(d4T + 3TC + EFV), 1c(AZT + 3TC + NVP), 1d(AZT + 3TC + EFV), 1e(TDF + 3TC + EFV), 1f(TDF + 3TC + NVP)	4809	113
Hailu et al. [42]	2018	Retrospective follow-up study	Ethiopia	Adults (≥ 20 years)	TDF 3TCEFV/NVP, AZT 3TC NVP/EFV, D4T 3TC NVP/EFV, ABC 3TC EFV	260	30
Hassan et al. [14]	2014	Cross-sectional study	Kenya	Adult	Zidovudine-based and Stavudine-based	232	57
Izudi et al. [43]	2016	Retrospective cohort	Uganda	Adult		383	28
Karade et al. [44]	2016	Cross-sectional studies	India	Adult	AZT + 3TC + NVP, AZT + 3TC + EFV TDF + 3TC + NVP, TDF + 3TC + EFV d4T + 3TC + NVP/EFV	844	104
Lay et al. [45]	2017	Retrospective cohort study	Cambodia	Adult (≥ 18 years old)	d4T/3TC/EFV, d4T/3TC/NVP AZT/3TC/EFV, AZT/3TC/NVP Other	3581	137
Ndahimana et al. [46]	2016	Retrospective cohort	Rwanda	15 years and older	NRTIs, NNRTIs, and PIs	828	70
Ahmed et al. [47]	2019	Case-control study	Ethiopia	Adult	d4t + 3TC + NVP, AZT + 3TC + NVP AZT + 3TC + EFV, TDF + 3TC + EFV TDF + 3TC + NVP	308	199

### Meta-analysis

**Fig. 2:**
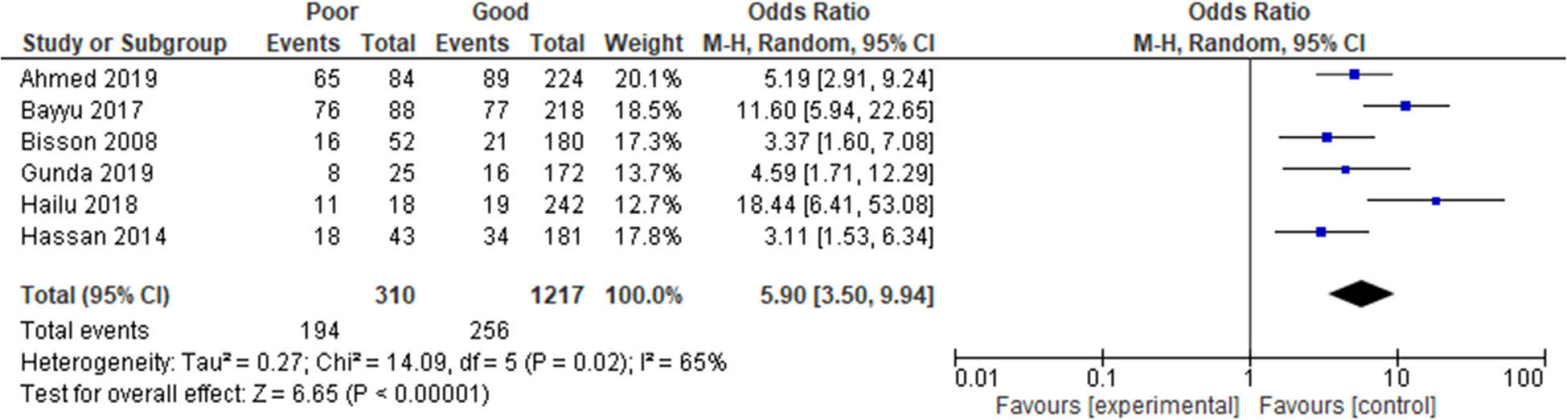
Pooled odds ratio between adherence and treatment failure. Comparison: poor versus good adherence (outcome: virological failure)

The association between adherence and treatment failure was based on six cross-sectional studies [14, 35, 37, 40, 42, 47]. The results as presented in Fig. 2 showed a strong relationship between treatment failure and poor treatment adherence. The odds of treatment failure were nearly 6 times higher among patients who had poor adherence (OR = 5.90, 95% CI 3.50, 9.94, moderate strength of evidence). The test statistics, however, showed a substantial heterogeneity (*I*^2^ = 65% and *p* = 0.02).

**Fig. 3:**
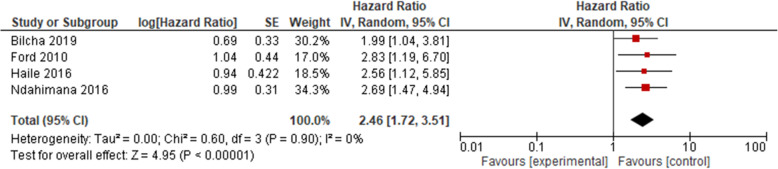
Pooled odds ratio between adherence and treatment failure. Comparison: poor versus good adherence (outcome: virological failure)

Similarly, the association between poor adherence and treatment failure was examined using four cohort studies [36, 39, 41, 46]. The results as presented in Fig. 3 showed that the hazard ratio of treatment failure was nearly 2.5 higher among patients who had poor adherence (HR = 2.46, 95% CI 1.72, 3.51, high strength of evidence). The result of test statistics showed no heterogeneity (*I*^2^ = 0% and *p* = 0.90). Here too, a random effect meta-analysis model was used to determine the association with the outcome.

**Fig. 4:**
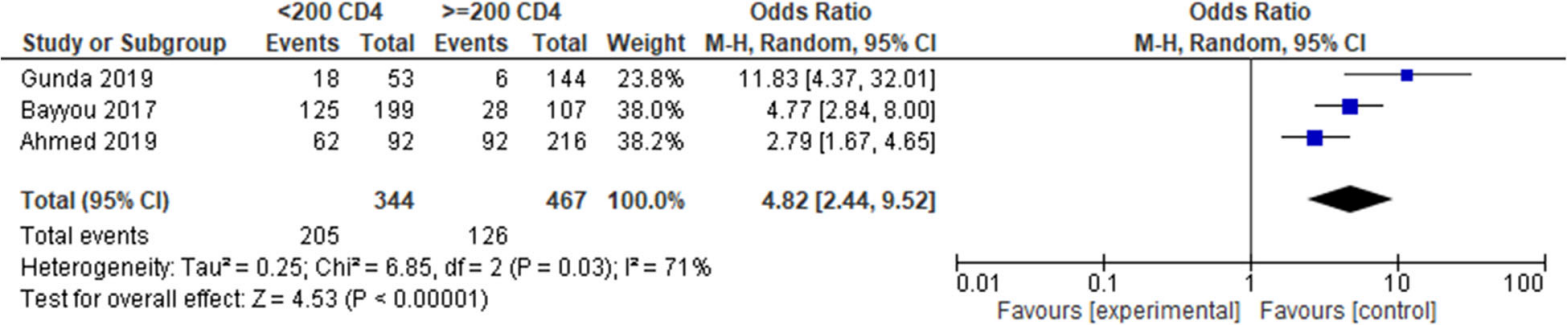
Pooled odds ratio between CD4 and treatment failure. Comparison: CD4 < 200 cells/mm^3^ versus CD4 ≥ 200 cells/mm^3^ (outcome: virological failure)

Furthermore, the association between CD4 and treatment failure was examined by using three cross-sectional studies [35, 40, 47]. The results as presented in Fig. 4 showed that treatment failure was strongly associated with CD4 count. The odds of treatment failure were nearly 5 times higher among patients who had a CD4 cell count of 200 cells/mm^3^ (OR = 4.82, 95% CI 2.44, 9.52, low strength of evidence). However, the test statistics showed substantial heterogeneity (*I*^2^ = 71% and *p* = 0.03). Hence, a random effect meta-analysis model was used to determine the association with the outcome.

**Fig. 5:**
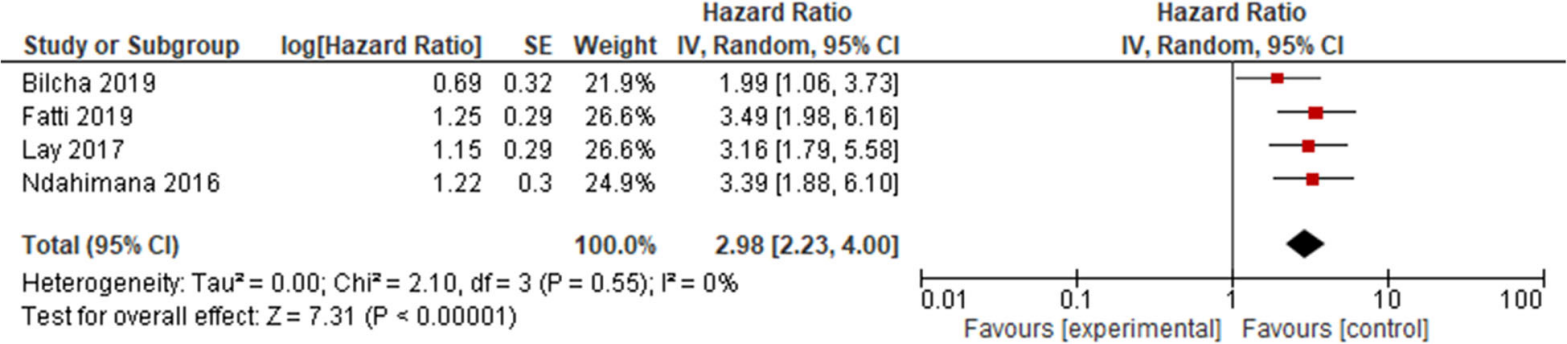
Pooled odds ratio between CD4 and treatment failure. Comparison: CD4 < 200 versus CD4 ≥ 200 (outcome: virological failure)

Likewise, the association between low CD4 count and treatment failure was also observed using four cohort studies [36, 38, 45, 46]. Results presented in Fig. 5 showed that the hazard ratio of treatment failure was nearly 3 times higher among patients who had CD4 lower than 200 cells/mm^3^ (HR = 2.98, 95% CI 2.23, 4.00, moderate strength of evidence). The result of the test statistics showed no evidence of heterogeneity (*I*^2^ = 0% and *p* = 0.55). A random effect meta-analysis model was used to determine the association with the outcome.

**Fig. 6:**
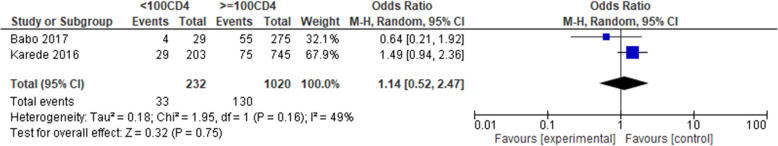
Pooled odds ratio between CD4 and treatment failure. Comparison: CD4 < 100 versus CD4 ≥ 100. (outcome: virological failure)

Our study also demonstrated similar findings to the above through data abstracted from two cross-sectional studies [34, 44]. We also found that treatment failure was significantly associated with low CD4 count, where the odds of treatment failure were 1.14 times higher among patients with CD4 lower than 100 cells/mm^3^ (OR = 1.14, 95% CI 0.52, 2.47, low strength of evidence). The test statistics showed moderate heterogeneity (*I*^2^ = 49% and *p* = 0.75), see Fig. 6. Consequently, a random effect meta-analysis model was computed to determine the association.

### Risk of bias assessment

Most of the studies had a low risk of bias on prognostic factors that accounted for 125/137, followed by study participants (123/135), statistical analysis and reporting (116/137), and outcome measurement (115/137). Moreover, 109/137 studies had a low risk of bias on study confounding and 103/137 studies had a low risk of bias on study participant attrition. The full table of results is shown in Appendix 3: risk of bias assessment.

## Discussion

This review was aimed at identifying factors associated with antiretroviral treatment failure among individuals living with HIV and showed that low CD4 T cell count (≤ 200 cells/mm^3^) and poor adherence to ART were significantly associated with virological failure.

In this review, the odds of virological failure were higher among those who had a CD4 cell count of ≤ 200cells/mm^3^ in both case-control and cohort studies. The finding is supported by the studies conducted in SSA [35, 43], while a retrospective analysis of a large ART program in Cambodia showed that previous ART experience, nevirapine-based regimen, and CD4 count ≤ 200 cells/mm^3^ were independently associated with an increased risk of treatment [48]. Similar findings were reported in a meta-analysis data from India, where CD4 count ≤ 200 had a significantly greater risk of treatment failure [49]. As CD4 cell count increases, viral replication decreases, which means it has an inverse relationship with viral load. As patients’ immune status drops, and the rate of viral load increases compared to the immuno-competent individuals with HIV infection. In addition, users with compromised immunity are more susceptible to different opportunistic infections that endure the cruel cycle of immunity depletion and viral replication [50].

Moreover, the results found from case-control studies shown that the odds of virological failure were 6 times more among those who had poor adherence compared with those who had good adherence to antiretroviral treatment. Likewise, the finding from cohort studies showed that the odds of virological failure were higher among those who had poor adherence compared with those who had good adherence to antiretroviral treatment. This finding is supported by findings from primary studies conducted in African countries [11, 51, 52], but also consistent with the finding from a study conducted in Vietnam and other developed countries [53–55]. It is obvious that poor adherence to medication compromises treatment response due to suboptimal drug concentration hence creates a conducive environment for viral replication leading to virological failure [56, 57]. This reaffirms the need for reinforcement of drug adherence counseling for HIV patients before and during their life course of taking ART.

Poor adherence may lead to a number of adverse consequences on both individual and public HIV healthcare levels. Therefore, the measured efforts are immediately needed in HIV care by responsive bodies like ART case managers, adherence counselors in the hospitals on patients with low current CD4 count through improving poor adherence to ART treatment by strengthening enhanced adherence counseling. Each low-income country national HIV program should give attention to improving HIV services to strengthen adherence among patients on ART in order to reduce the proportion of patients who are failing the treatment.

Our systematic review has some strengths. We planned the review a priori with clearly defined selection criteria. We conducted a comprehensive and exhaustive search, using many additional sources to identify relevant studies, including reference searches of other HIV/AIDS conferences (IAS and CROI) for the past 20 years.

This review had several limitations mainly related to the quality of the evidence available. To our knowledge, we suspect publication or reporting biases, or both, suggesting that our results may be overestimated. Positive study bias is likely to be problematic in this review. Our literature search for relevant and potential studies included focused searches, i.e., including search terms related to the “less CD4 count,” “viral load” in our electronic search. Studies that report a relationship between the prognostic factors and common outcomes are therefore more likely to have been identified in these searches due to reporting of positive results in the study abstract.

In addition, we also observed that some studies reported positive unadjusted association of factors with outcomes of interest, but did not report the association adjusted for other important covariates. This may contribute to a likely overestimation of the adjusted results. Therefore, future research is required to investigate the impact and potential strategies to alleviate reporting and publication bias, as well as initiatives to require registration of protocols and publication of prognostic studies.

Furthermore, our review was the pooling of the adjusted the results despite studies did not include identical sets of covariates. Studies included in this review were homogenous; therefore, pooling of the adjusted results was feasible. However, comparison and interpretation may be challenging in this case. Our review only focused on studies conducted in poor resource settings limiting its generalizability to high-income settings.

### Strength of evidence

The strength of evidence contributing to several outcomes in this review was graded as low, moderate, or high. We used the GRADE approach to assess the strength of evidence as shown in the summary of the finding table, Appendix 4. The certainty of evidence was downgraded in most instances due to a high risk of bias as well as inconsistency.

## Conclusion

ART failure among individuals living with HIV is a public health concern; the timing and accuracy of identifying treatment failure in resource-limited settings are fundamental but challenging. The findings of this review highlighted that low CD4 counts and poor adherence to ART were associated to ART treatment failure. There is an urgent need that health professionals and HIV programs should focus on novel approaches for patients who have these characteristics in order to prevent ART failure. Further review is required to be done in multiple ART centers and a broader community as well as the different factors associated with treatment failure to decide whether there are discrepancies in virological and immunological responses to antiretroviral therapy at different stages of HIV infection.

## Data Availability

All data generated or analyzed during this study are included in this published article and its additional files.

## Appendix 1

### Search strategy—database

#1 Search ((HIV OR hiv-1 OR hiv-2* OR hiv1 OR hiv2 OR hiv infect* OR human immunodeficiency virus OR human immune deficiency virus OR human immuno-deficiency virus OR human immune-deficiency virus OR ((human immun*) AND (deficiency virus)) OR acquired immune deficiency syndromes

OR acquired immune deficiency syndrome OR acquired immuno-deficiency syndrome OR

acquired immune-deficiency syndrome OR ((acquired immun*) AND (deficiency syndrome)) OR HIV/AIDS))

#2 Search ((HIV infections [MeSH] OR HIV [MeSH]))

#3 Search (#1 OR #2)

#4 Search ((Antiretroviral* OR ((anti) AND (retroviral*)) OR ARV* OR ART OR “antiretroviral therapy”

OR HAART OR ((highly) AND (active) AND (antiretroviral*) AND (therap*)) OR ((anti) AND (hiv)) OR

((anti) AND (acquired immunodeficiency)) OR ((anti) AND (acquired immuno-deficiency)) OR ((anti)

AND (acquired immune-deficiency)) OR ((anti) AND (acquired immun*) AND (deficienc*))))

#5 Search ((antiretroviral agents [Mesh] OR antiretroviral therapy, highly active [Mesh]))

#6 Search (#4 OR #5)

#7 search #3 AND #6

#8 Search (virological failure OR Immunological failure OR less CD4 count OR viral load)

#9 Search (low-income setting OR disadvantaged communities OR resource limited setting OR Sub-Saharan Africa)

#10 Search (#7 AND #8 AND #9)

## Appendix 2

### Risk of bias criteria and justifications

**Table:**

**Assessment for risk of bias**			
First author	Reviewer........................		
**Biases**	**Issues to consider for judging overall rating of “risk of bias”**	**Study methods and comments**	**Rating of risk of bias**
Assess the risk of each potential bias	These issues will guide your thinking and judgment about the overall risk of bias within each of the 6 domains.	Provide comments or excerpts to facilitate the consensus process that will follow	**High, moderate, low**
**1) Study participation**	The study sample adequately represents the population of interest		**Summary**
a. Adequate participation in the study by eligible persons (> 80%)			**High bias:** The relationship between the PF and outcome is very likely to be different for participants and eligible nonparticipants **Moderate bias:** The relationship between the PF and outcome may be different for participants and eligible nonparticipants **Low bias:** The relationship between the PF and outcome is unlikely to be different for participants and eligible nonparticipants
b. Description of the source population or population of interest			
c. Description of the baseline study sample			
d. Adequate description of the sampling frame and recruitment.			
e. Adequate description of the period and place of recruitment			
f. Adequate description of inclusion and exclusion criteria			
**2) Study attrition**	The study data available (i.e., participants not lost to follow-up) adequately represent the study sample		**Summary**
a. Adequate response rate for study participants *(> 80%)*			**High bias**: The relationship between the PF and outcome is very likely to be different for completing and noncompeting participants **Moderate bias**: The relationship between the PF and outcome may be different for completing and noncompeting participants **Low bias**: The relationship between the PF and outcome is unlikely to be different for completing and noncompeting participants
b. Description of attempts to collect information on participants who dropped out			
c. Reasons for loss to follow-up are provided			
d. Adequate description of participants lost to follow-up			
e. There are no important differences between participants who completed the study and who did not			
**3) Prognostic factor measurement**	The PF is measured in a similar way for all participants		**Summary**
a. A clear definition or description of the PF is provided			**High bias**: The measurement of the PF is very likely to be different for different levels of the outcome of interest **Moderate bias**: The measurement of the PF may be different for different levels of the outcome of interest **Low bias**: The measurement of the PF is unlikely to be different for different levels of the outcome of interest
b. Method of PF measurement is adequately valid and reliable (i.e., direct ascertainment; secure record, hospital record)			
c. Continuous variables are reported or appropriate cut-points are used			
d. The method and setting of measurement of PF is the same for all study participants			
e. Adequate proportion of the study sample has complete data for the PF *(> 80%)*			
f. Appropriate methods of imputation are used for missing PF data			
**4) Outcome measurement**	The outcome of interest is measured in a similar way for all participants		**Summary**
a. A clear definition of the outcome of interest is provided (including the time of death)			**High bias**: The measurement of the outcome is very likely to be differently related to the baseline level of the PF **Moderate bias**: The measurement of the outcome may be differently related to the baseline level of the PF **Low bias**: The measurement of the outcome is unlikely to be differently related to the baseline level of the PF
b. Method of outcome measurement used is adequately valid and reliable (i.e. independent blind assessment, hospital record or record linkage)			
c. The method and setting of outcome measurement is the same for all study participants			
**5) Study confounding**	Important potential confounder is appropriately accounted for		**Summary**
a. *Most* important confounders are measured			**High bias:** The observed effect of the PF on the outcome is very likely to be distorted by another factor related to PF and outcome **Moderate bias:** The observed effect of the PF on outcome may be distorted by another factor related to PF and outcome **Low bias:** The observed effect of the PF on the outcome is unlikely to be distorted by another factor related to PF and outcome
b. Clear definitions of the important confounders measured are provided			
c. Measurement of all important confounders is adequately valid and reliable			
d. The method and setting of confounding measurement are the same for all study participants			
e. Appropriate methods are used if imputation is used for missing confounder data			
f. Important potential confounders are accounted for in the study design (by limiting the study to specific population groups, or by matching)			
g. Important potential confounders are accounted for in the analysis *(by stratification, multivariate regression)*			
**6) Statistical analysis and presentation**	The statistical analysis is appropriate, and all primary outcomes are reported		**Summary**
a. Sufficient presentation of data to assess the adequacy of the analytic strategy			**High bias**: The reported results are very likely to be spurious or biased related to analysis or reporting **Moderate bias**: The reported results may be spurious or biased related to analysis or reporting **Low bias**: The reported results are unlikely to be spurious or biased related to analysis or reporting
b. Strategy for model building is appropriate and is based on a conceptual framework or model			
c. The selected statistical model is adequate for the design of the study			
d. There is no selective reporting of results (*based on the study protocol, if available, or on the “Methods” section*)			

## Appendix 3

### Risk of bias assessment

**Table 2:** Risk of bias assessment

#	Study ID	Study		Prognostic			Statistical analysis and reporting
		participant	Attrition	Factor measurement	Outcome measurement	Study confounding	
1	Abah 2018	Low	High	Low	Low	Low	Low
2	Ahmed 2019	Low	Low	Low	Low	Low	Low
3	Ahn 2019	Low	High	Low	Low	Low	Low
4	Ahoua 2009	Low	Low	Low	Low	Low	Low
5	Assefa 2014	Low	High	Low	Low	Low	Low
6	Ayalew 2016	Low	Low	Low	Low	Low	Low
7	Ayele 2018	Low	Low	Low	Low	Low	Low
8	Babo 2017	Low	Low	Low	Low	Low	Low
9	Bayou 2015	Low	High	Low	Low	Low	Low
10	Bayu 2017	Low	Low	Low	Low	Low	Low
11	Billioux 2015	Low	Low	High	High	Low	Low
12	Biscione 2014	Low	Low	High	High	High	Low
13	Bisson 2008	Low	Low	High	Low	High	Low
14	Boender 2016a	Low	Low	Low	Low	Low	Low
15	Boender 2016b	Low	Low	Low	Low	Low	Low
16	Boettiger 2016c	Low	Low	Low	Low	Low	Low
17	Boettiger 2015	Low	Low	Low	Low	Low	Low
18	Boettiger 2016d	Low	Low	Low	Low	Low	Low
19	Boettiger 2014	Low	Low	Low	Low	Low	Low
20	Boulle 2015	Low	Low	Low	Low	Low	Low
21	Braun 2017	Low	Low	Low	Low	High	High
22	Brooks 2016	Low	Low	Low	Low	High	High
23	Bulage 2017	Low	High	Low	Low	Low	Low
24	Byabene 2017	Low	Low	Low	Low	Low	Low
25	Cao 2018	Low	Low	Low	Low	Low	Low
26	Carriquiry 201	Low	Low	Low	Low	Low	Low
27	Caseiro 2018	Low	Low	Low	High	High	Low
28	Castelnuovo 2016	Low	Low	Low	Low	Low	Low
29	Cesar 2015	Low	Low	Low	Low	Low	Low
30	Cesar 2014	Low	Low	Low	Low	Low	Low
31	Chaiwarith 2011	Low	Low	Low	Low	Low	Low
32	Chaiwarith 2007	Low	Low	High	Low	Unclear	Low
33	Chakravarty 2015	Low	Low	Low	Low	Unclear	Low
34	Charles 2013	Low	Unclear	Low	Low	Unclear	Low
35	Chawana 2014	Low	Low	Low	Low	Unclear	Low
36	Chen 2014	Low	High	Low	Low	High	Low
37	Chhim 2018	Low	High	Low	Low	Low	Low
38	Chkhartishvili 2014	Low	Low	Low	Low	Unclear	Low
39	Collier 2017	Low	Low	Low	Low	Low	Low
40	Costiniuk 2014	High	Unclear	Low	Low	High	High
41	Court 2014	Low	Low	Low	Low	Low	Low
42	Datay 2010	Low	Low	Low	Low	Unclear	Low
43	DeBoni 2018	Low	Low	Low	Low	Low	Low
44	deLaHoz 2014	Low	Low	Unclear	Unclear	Unclear	Low
45	Dolling 2017	Low	Low	Low	Low	Low	Low
46	Dray-Spira 2007	Low	Low	Low	Low	Unclear	Low
47	Ekstrand 2011	Low	Low	High	Unclear	Unclear	High
48	Rusine 2013	Low	Low	Low	Low	Low	Low
49	Sadashiv 2017	Low	Low	Low	Low	Low	Low
50	Safren 2014	Low	Low	Low	Low	Low	Low
51	Saracino 2014	Low	Low	Low	Low	Low	Low
52	Singini 2016	Low	Low	Low	Low	Low	Low
53	Sithole 2018	Low	Low	Low	Low	Low	Low
54	Sovershaeva 2019	Low	Low	Low	Low	Low	Low
55	Syed 2016	Low	Low	Low	Low	Low	Low
56	Telele 2018	Low	Low	Low	Low	Low	Low
57	Teshome 2014	Low	Low	Low	Low	Low	Low
58	Thiha 2016	High	Low	Low	Low	Low	Low
59	Tran 2014	Low	High	Low	Low	Low	Low
60	Tsegaye 2016	Low	High	Low	Low	Low	Low
61	vandenBerg 2005	Low	Low	Low	Low	Low	Low
62	Vanobberghen 2015	Low	Low	Low	Low	Low	Low
63	Wang 2011	High	High	Low	Low	Low	Low
64	Yimer 2015	Low	Low	Low	Low	Low	Low
65	Yirdaw 2015	Low	Low	Low	Low	Low	Low
66	Zhao 2017	Low	High	Low	Low	Low	Low
67	Zoufaly 2015	Low	Low	Low	Low	Low	Low
68	Elema 2009	Low	Low	Unclear	low	Unclear	Low
69	Enderis 2009	Low	Low	Low	Low	Low	Low
70	Eshleman 2017	Low	Low	Low	Low	Low	Low
71	Evans 2018	Low	High	Low	Low	Low	Low
72	Evans 2013	Low	High	Low	Low	Low	Low
73	Fatti 2019	Low	Low	Low	Low	Low	Low
74	Fatti 2014	Low	Low	Low	Low	Low	Low
75	Ferradini 2007	Low	Low	Low	Low	Low	Low
76	Ferreyra 2012	Low	Low	Low	Low	Low	Low
77	Fibriani 2013	Low	Low	Low	Unclear	Unclear	Low
78	Flynn 2017	Low	Low	Low	Low	Low	Low
79	Fogel 2017	unclear	Unclear	Low	Low	Low	Unclear
80	Ford 2010	Low	Low	Low	Low	Low	Low
81	Fox 2012	Low	Low	Low	Low	Low	Low
82	Fox 2010	Low	Low	Low	Low	Low	Low
83	Goldman 2008	Low	Low	Low	Low	Unclear	Low
84	Gross 2017	Low	Low	Low	Low	Low	Low
85	Gunda 2019	Low	Low	Low	Low	Low	Low
86	Haggblom 2016	Low	Low	Low	Low	Low	Low
87	Haile 2016	Low	High	Low	Low	High	Low
88	Hailu 2018	Low	Low	Low	Low	Low	Low
89	Hamers 2012	Low	Low	Low	Low	Low	Low
90	Hare 2014	Low	Low	Low	Low	Low	Low
91	Hassan 2014	Low	Low	Low	Low	Low	Low
92	Hawkins 2015	Low	High	Low	Low	Low	Low
93	Hawkins 2016	Low	Low	Low	Low	Low	Low
94	Hermans 2018	Low	High	Unclear	Low	Unclear	Low
95	Huang 2015	Low	High	Low	Low	Low	Low
96	Hunt 2017	Low	Low	Low	High	Unclear	Low
97	Huong 2011	Low	Low	Low	low	Unclear	Low
98	Inzaule 2018	Low	Low	Unclear	low	Low	Low
99	Izudi 2016	Low	Low	Unclear	low	Low	Low
100	Jiamsakul 2016	Low	High	Low	low	Low	Low
101	John 2016	Low	Low	Low	low	Low	Low
102	Joram 2017	Low	High	Low	High	Low	Low
103	JosephDavey 2018	Low	Low	Low	low	Low	Low
104	Kamya 2007	Low	Low	Unclear	low	Low	Low
105	Kan 2017	Low	Low	Low	High	Low	Low
106	Karade 2016	Low	Low	Low	High	Low	Low
107	Kazooba 2018	Low	High	Low	Low	Low	Low
108	Khienprasit 2011	Low	Low	Low	High	Low	High
109	Kyaw 2017	Low	High	Low	High	Low	Low
110	Lay 2017	Low	Low	Low	High	Low	High
111	Leng 2014	Low	Low	High	High	Low	Low
112	Lenjisa 2015	Low	High	Low	Low	Low	High
113	Levison 2011	Low	Low	Low	Low	High	High
114	Liegeois 2013	Low	Low	Low	High	Low	High
115	Masikini 2019	High	Low	Low	High	Low	Low
116	Meloni 2016	Low	Low	Low	High	High	Low
117	Mpawa 2017	High	High	Low	High	Low	High
118	Mujugira 2016	Low	Low	Low	low	Low	Low
119	Mungwira 2018	Low	High	Low	High	Low	Low
120	Musa 2015	Low	Low	Low	Low	Low	Low
121	Nachega 2008	High	Low	Low	Low	High	Low
122	Ndahimana 2016	High	Low	Low	Low	Low	High
123	Negi 2018	Low	High	Low	Low	Low	HIgh
124	Nsanzimana 2019	High	High	Low	Low	Low	High
125	Ntamatungiro 2017	Low	High	Low	Low	Low	High
126	Ongubo 2017	High	High	Low	Low	Low	High
127	Onoya 2016	Low	High	Low	Low	Low	Low
128	Palladino 2013	High	Low	Low	Low	Low	Low
129	Patrikar 2017	Low	Low	Low	High	Low	High
130	Penot 2014	High	Low	Low	High	Low	High
131	Raimondo 2017	Low	Low	Low	low	Low	Low
132	Rajasekaran 2007	Low	Low	Low	High	High	Low
133	Ramadhani 2007	High	Low	Low	low	Low	High
134	Rangarajan 2016	Low	High	Low	low	Low	Low
135	Rohr 2016	Low	High	Low	low	Unclear	Low
136	Ruperez 2014	High	Low	Low	low	Low	High
137	Ruperez 2015	High	Low	Low	low	Low	High

## Appendix 4

### Strength of evidence

**Table 3:** Summary of findings of included studies using the GRADE methodology (Grading of Recommendations Assessment, Development and Evaluation)

Factors assessed	Number of studies (SD)	Main findings	Strength of evidence (high, moderate, low, very low)
Adherence (poor versus good)	6 (cross-sectional)	Odds ratio: 5.90 (95%CI, 3.50–9.94)	Moderate^a^
Adherence (poor versus good)	4 (cohort studies)	Hazar ratio: 2.46 (95% CI, 1.72–3.51)	High
CD4 cell count (< 200 versus ≥ 200 cells/mm^3^)	3 (cross-sectional)	Odd ratio: 4.82 (95% CI, 2.44–9.52)	Low^b^
CD4 cell count (< 200 versus ≥ 200 cells/mm^3^)	4 (cohort studies)	Hazard ratio: 2.98 (95% CI, 2.23–4.0)	Moderate^c^
CD4 cell count (< 100 versus ≥ 100 cells/mm^3^)	2 (cross-sectional)	Odds ratio: 1.14 (95% CI, 0.52–2.47)	Low^d^

*SD* study design

^a^Downgraded once to indirectness, the final sample of some of the included studies only represents the population of interest

^b^Imprecision and inconsistency were major concerns, imprecision due to a limited number of studies and wide confidence intervals, and there was a substantial heterogeneity statistical heterogeneity (heterogeneity: Tau^2^ = 0.25; chi^2^ = 6.25, df = 2(*P* = 0.03), *I*^2^ = 71%) and marked clinical heterogeneity

^c^Downgraded once due to a risk of bias, bias to statistical analysis and reporting, and potential confounding factors

^d^Imprecision due to a limited number of participants and studies included. Inconsistency as there was a moderate statistical heterogeneity (heterogeneity: Tau^2^ = 0.18; chi^2^ = 1.95, df = 1 (*P* = 0.16); *I*^2^ = 49%)

GRADE Working Group grades of evidence

High certainty: We are very confident that the true effect lies close to that of the estimate of the effect

Moderate certainty: We are moderately confident in the effect estimate: The true effect is likely to be close to the estimate of the effect, but there is a possibility that it is substantially different

Low certainty: Our confidence in the effect estimate is limited: The true effect may be substantially different from the estimate of the effect

## Abbreviations

AIDS: Acquired immune deficiency syndrome
ART: Antiretroviral therapy
BMI: Body mass index
CDC: Centers for Disease Control and Prevention
CENTRAL: Cochrane Central Register of Controlled Trials
EMBASE: Excerpta Medica Database
HRs: Hazard ratios
HIV: Human immunodeficiency virus
IRIS: Immune reconstitution inflammatory syndrome
LILACS: Latin American and Caribbean Health Sciences Literature
LMICs: Low- and middle-income countries
MEDLINE: Medical Literature Analysis and Retrieval System Online
NNRTI: Non-nucleotide reverse transcriptase inhibitors
NRTI: Nucleotide reverse transcriptase inhibitors
OR: Odds ratio
PLHIV: People living with human immunodeficiency virus
PRISMA-P: Preferred Reporting Items for Systematic Reviews and Meta-Analysis Protocols
PROSPERO: Prospective Register of Systematic Reviews
PubMed: Public/Publisher MEDLINE
RRs: Risk ratios
SSA: Sub-Saharan Africa
TB: Tuberculosis
UNAIDS: United Nations Programme on HIV
WHO: World Health Organization
